# Comparison of Rumen and Manure Microbiomes and Implications for the Inoculation of Anaerobic Digesters

**DOI:** 10.3390/microorganisms6010015

**Published:** 2018-02-14

**Authors:** Emine Gozde Ozbayram, Orhan Ince, Bahar Ince, Hauke Harms, Sabine Kleinsteuber

**Affiliations:** 1 Department of Environmental Engineering, Faculty of Civil Engineering, Istanbul Technical University, Maslak, 34469 Istanbul, Turkey; gozbayram@itu.edu.tr (E.G.O.); inceor@itu.edu.tr (O.I.); 2 Department of Environmental Microbiology, Helmholtz Centre for Environmental Research—UFZ, 04318 Leipzig, Germany; hauke.harms@ufz.de; 3 Institute of Environmental Sciences, Boğaziçi University, Bebek, 34342 Istanbul, Turkey; bahar.ince@boun.edu.tr

**Keywords:** ruminant microbiome, plant fiber fermentation, anaerobic digestion, bioaugmentation, hydrolytic bacteria, methanogenic archaea, *mcrA* gene, 16S rRNA gene, 454 amplicon sequencing

## Abstract

Cattle manure is frequently used as an inoculum for the start-up of agricultural biogas plants or as a co-substrate in the anaerobic digestion of lignocellulosic feedstock. Ruminal microbiota are considered to be effective plant fiber degraders, but the microbes contained in manure do not necessarily reflect the rumen microbiome. The aim of this study was to compare the microbial community composition of cow rumen and manure with respect to plant fiber-digesting microbes. Bacterial and methanogenic communities of rumen and manure samples were examined by 454 amplicon sequencing of bacterial 16S rRNA genes and *mcrA* genes, respectively. Rumen fluid samples were dominated by Prevotellaceae (29%), whereas Ruminococcaceae was the most abundant family in the manure samples (31%). Fibrobacteraceae (12%) and Bacteroidaceae (13%) were the second most abundant families in rumen fluid and manure, respectively. The high abundances of fiber-degrading bacteria belonging to Prevotellaceae and Fibrobacteraceae might explain the better performance of anaerobic digesters inoculated with rumen fluid. Members of the genus *Methanobrevibacter* were the predominant methanogens in the rumen fluid, whereas methanogenic communities of the manure samples were dominated by the candidate genus *Methanoplasma*. Our results suggest that inoculation or bioaugmentation with fiber-digesting rumen microbiota can enhance the anaerobic digestion of lignocellulosic biomass.

## 1. Introduction

During the last decade, demand for livestock products, particularly in developing countries, has massively grown as a result of population growth, urbanization, and rising income [1]. The increasing number and size of animal production plants creates the need for better waste management in livestock farming. Biogas production from crop residues and animal manure by anaerobic digestion is a sustainable approach for waste reduction and energy recovery. Hydrolysis is considered as the rate-limiting step during the anaerobic digestion of these waste streams due to their high content of lignocellulosic materials. Consequently, numerous studies have focused on the development of feedstock pretreatment methods and inoculation strategies in order to improve the hydrolytic efficiency and consequently enhance the rates of acidogenesis and methanogenesis. 

Ruminants have unique digestive systems bearing on a symbiotic relationship between bacteria, archaea, protozoa, and fungi within the rumen environment [2,3]. Plant polymers such as cellulose, hemicellulose, and lignin components are degraded by hydrolytic exoenzymes released or exposed by microbes and subsequently converted by acidogenic fermentation into short chain fatty acids, which are consumed as nutrient by the ruminant. Thus, ruminants can utilize plant material more effectively and conserve more of its energy than other herbivores, which is due to the dense and diverse microbial consortia in the rumen that carry out these metabolic activities [4]. Besides bacterial communities, the rumen microbiota includes methanogens which utilize H_2_ and CO_2_ to produce methane and reduce hydrogen accumulation [5]. The fermentation of plant materials in the ruminant digestive tract and environmental conditions in the rumen, such as pH, temperature, and redox potential, resemble the anaerobic digestion process in a biogas reactor, at least concerning the first steps of the process (hydrolysis and acidogenesis) and hydrogenotrophic methanogenesis [6]. The use of rumen fluid as an inoculum for anaerobic digesters fed with plant fibers is thus an approach that has gained increasing attention in recent years. Inoculum selection is of major importance in the start-up period of anaerobic digesters and the use of rumen fluid promises to increase the decomposition of plant fibers [7,8,9]. Though cattle manure is a suitable substrate for anaerobic digesters, its recalcitrant fiber structure and high water content results in a low methane yield in mono-digestion. The combination of different substrates with manure facilitates the digestion by increasing the easily degradable fraction and thus enhancing the methane yield [10]. Cattle manure is commonly considered as an inoculum well-suited for the start-up of anaerobic digesters, since it contains a diverse microbial community that can easily adapt to changing operational conditions [11,12]. Most studies on the rumen environment focused on microbial community dynamics depending on the dietary differentiation of animals [3,13,14]. However, the microbial diversity and especially possible differences between the structures of microbial communities in the rumen and the manure of the same animal has not yet received much attention. This knowledge, however, is required to identify the key microorganisms of use for efficient lignocellulose degradation. 

The microbial community composition of the rumen environment had been studied primarily by cultivation-based methods for many decades until the 1980s, when these techniques were replaced by molecular tools which facilitate the study of complex environments [15]. Recently, a considerable literature on the microbial community composition of the rumen ecosystem arose thanks to the development of precise molecular techniques [3,4,13,16,17,18,19,20,21]. However, only a few attempts have been made to compare the rumen and manure microbial communities [20].

The aim of this study was to determine the composition of the microbiota in cow rumen and manure to reveal similarities and differences between these two communities. Microbial resources from these environments might help improve the efficiency of anaerobic digesters treating lignocellulosic feedstocks. 

## 2. Materials and Methods

Three mature, non-medicated healthy Jersey cows (average body weight 450 kg) used in this study were cared for and handled in a barn of the Veterinary Faculty of Leipzig University, Germany. All cows were fed the same diet, which consisted of 81% hay, 11% concentrated corn with minerals and vitamins, and 8% soybean meal (based on dry matter). Rumen fluid was sampled via rumen fistulae and feces were sampled from the rectum according to institutional animal care guidelines. From each cow, 50 mL grab samples were taken and kept at 38 °C during transfer to the lab. Afterwards, samples were aliquoted in Eppendorf tubes, frozen immediately, and stored at −80 °C until DNA extraction. DNA was extracted from 400 µL aliquots using the NucleoSpin Kit for Soil (Macherey-Nagel, Düren, Germany). Amplicon sequencing of bacterial 16S rRNA genes was done as described previously [22]. Methanogenic communities were analyzed based on *mcrA* amplicon sequencing using the primers mlas and mcrA-rev [23]. Sequence data were analyzed with the QIIME pipeline (Quantitative Insights Into Microbial Ecology; http://qiime.org) as described previously [24,25]. The bacterial and methanogenic community profiles were visualized by Krona graphs [26]. Venn diagrams were prepared to show the number of shared and unique operational taxonomic units (OTUs) between the samples by using Venny 2.1.0 [27]. Non-metric multidimensional scaling (NMDS) plots based on the Bray-Curtis dissimilarity index were calculated as described previously [21].

Sequence data were submitted to the European Nucleotide Archive (ENA) under the accession numbers PRJEB19600, PRJEB19601, and PRJEB19525.

## 3. Results and Discussion

### 3.1. Bacterial Communities of Rumen and Manure

**Figure 1:**
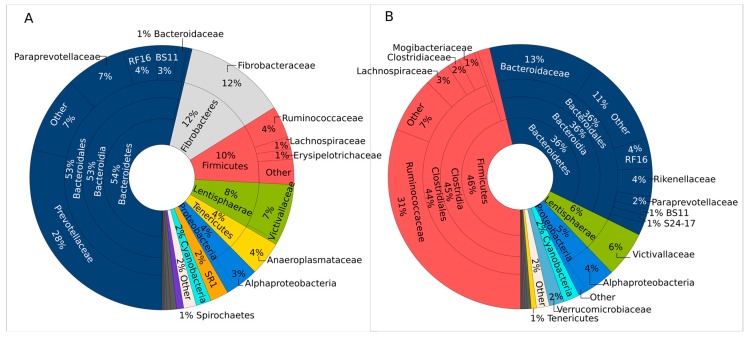
Krona charts illustrating the bacterial community composition of (**A**) the rumen samples and (**B**) the manure samples on phylum, class, order, and family levels. Sequence data from three individuals were combined.

The community compositions of rumen and manure samples as determined by 16S rRNA gene amplicon sequencing are visualized as Krona charts generated from the combined sequence dataset from the three animals (Figure 1). Krona graphs showing the individual community compositions are shown in the Supplementary Material (Figure S1). The rumen community (Figure 1A) was dominated by Bacteroidetes (54%), Fibrobacteres (12%), Firmicutes (10%), and Lentisphaerae (8%). Only 1.4% of the sequence reads could not be assigned to any bacterial phylum (unclassified bacteria). The remaining sequences were assigned to minor phyla, mainly Proteobacteria (4%), Tenericutes (4%), and candidate phylum SR1 (2%). The bacterial community of the manure sample (Figure 1B) was dominated by Firmicutes (46%), while Bacteroidetes was the second most abundant phylum (36%). The remaining phyla comprised Lentisphaerae (6%), Proteobacteria (5%), and Verrucomicrobia (2%), besides other minor phyla. Differently from rumen fluid, the abundance of the phylum Fibrobacteres was much lower in the manure samples (<0.1%), which was one of the main differences between the rumen and manure bacterial community profiles. As in the rumen samples, only a small portion (1%) of the reads could not be assigned to any bacterial phylum (unclassified bacteria).

In line with previous studies, the bacterial communities of both the rumen fluid and manure samples were dominated by the phyla Bacteroidetes and Firmicutes [3,19,20,21,28,29,30]. These findings are supported by previous studies employing clone libraries and Sanger sequencing [13,15]. Similar to our study, Liu and colleagues found a higher percentage of Firmicutes in feces than in rumen samples [20]. The changing ratios between the two major phyla show how the bacterial communities shift upon passing through the digestive tract of the ruminant. The phylum Fibrobacteres was much more abundant in our rumen samples compared to most previous studies, which found Fibrobacteres belonging to the cattle rumen core microbiome, but in lower amounts of less than 1% [3] or up to 3–4% [18]. On the one hand, this deviation might be due to a methodical bias such as different PCR primers or coverage of the different databases used for taxonomic assignment. On the other hand, it is known that the abundance of Fibrobacteres is affected by the fiber content of the diet. Several studies have recorded higher portions of this phylum in cattle fed a dry roughage diet [19] or in beef steers fed a higher percentage of hay compared to dairy cows, while including concentrate or starch and oil additives in the diet decreased the share of Fibrobacteres [3,4]. In accordance with our data, de Menezes et al. [21] detected up to 10% Fibrobacteres sequences in the solid phase of rumen content of dairy cows that were fed total mixed ration including straw. As we did not sieve our samples but included the particulate matter of rumen fluid in the analysis, this might further explain the high percentage of Fibrobacteres in our rumen samples.

At the family level, by far the greatest share of the sequences of the rumen fluid was assigned to Prevotellaceae (phylum Bacteroidetes; 28%). *Prevotella*, which belongs to the Prevotellaceae family, was the most dominant genus in the rumen fluid, coinciding with the results of previous studies on rumen microbiota [15,18,20,21]. Members of this genus play an important role in breaking down proteins and carbohydrates and represent the most numerous bacteria that are cultivable from the rumen and hindgut of cattle [20]. Moreover, some of the *Prevotella* species isolated from rumen produce cellulolytic enzymes like CMCase and xylanase [31]. Besides, it was suggested that *Prevotella* species may act synergistically with other cellulolytic organisms and are involved in ruminal fibrolytic activity [18,32]. In contrast, Prevotellaceae were not detected in the manure samples. 

In the manure samples, Ruminococcaceae was the predominant family (31%), which includes the genus *Ruminococcus.* Species of this genus were suggested to play a key role in ruminal cellulose decomposition [33]. However, the abundance of *Ruminococcus* in the rumen was much lower (4%) than that in the manure. This is in accordance with the study of Zened and colleagues, who found that less than 1% of the rumen bacterial community was contributed by the genus *Ruminococcus* [3]. The second most dominant families were Fibrobacteraceae (12%) in the rumen and Bacteroidaceae (13%) in the manure samples. Members of the Bacteroidaceae family have been described to ferment cellulosic material and produce acetic acid and hydrogen [34]. The family Fibrobacteraceae (phylum: Fibrobacteres) includes two cultured species, *Fibrobacter succinogenes* and *Fibrobacter intestinalis*, which have been suggested to be the main cellulose degraders in ruminant gut systems [35,36]. Wu et al. found a 570-fold increased abundance of *Fibrobacter* in mature rumen communities compared to the rumen of pre-ruminant calves, underpinning the importance of Fibrobacteraceae for plant-fiber digestion in cattle rumen [18].

**Figure 2:**
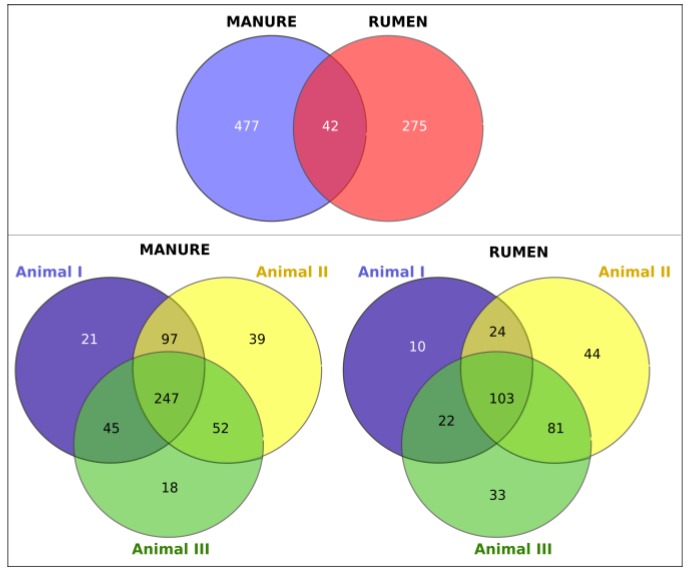
Venn diagrams of the bacterial communities showing the number of shared and unique operational taxonomic units (OTUs) in the rumen and manure samples.

The shared and unique OTUs between total rumen and manure samples and between the individual rumen and manure samples are presented in Figure 2. According to the Venn diagram in the upper panel, 722 OTUs were compartment-specific, whereas only 42 OTUs were detected in both rumen and manure samples. Comparing the individual animals, 247 OTUs were shared between all manure samples and 103 OTUs between all rumen samples, while only 78 and 87 OTUs, respectively, were individual-specific.

**Figure 3:**
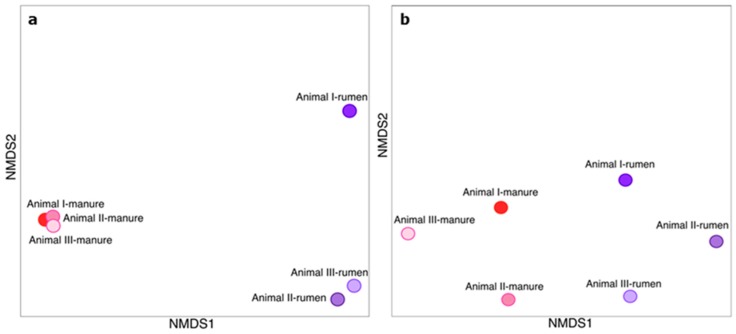
NMDS plots showing the Bray-Curtis dissimilarity of (**a**) the bacterial communities of rumen and manure samples based on 16S rRNA sequences and (**b**) the methanogenic communities of rumen and manure samples based on *mcrA* sequences.

As Venn diagrams show only the number of shared and unique OTUs regardless of their abundances, we compared the community profiles based on the Bray-Curtis dissimilarity index, which considers the presence/absence as well as relative abundance of OTUs. As illustrated in Figure 3a, the data points of the bacterial community profiles clustered according to the compartment but not to the individual, with a higher variance of the rumen samples compared to the manure samples. The higher variance of the manure bacterial communities compared to the rumen bacterial communities is also visible in the individual Krona graphs (Figure S1).

The estimated bacterial richness and evenness are shown in Table 1. The bacterial community of the manure was more diverse in terms of OTU number as well as Chao1 and Shannon indices compared to the rumen community. 

### 3.2. Methanogenic Communities of Rumen and Manure

**Figure 4:**
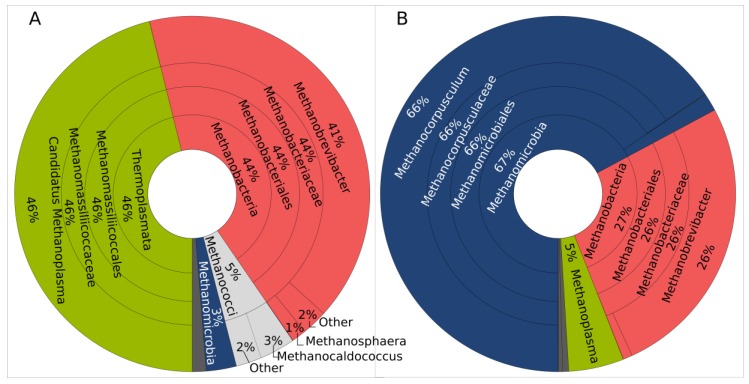
Krona charts illustrating the methanogenic community composition of (**A**) the rumen samples and (**B**) the manure samples on phylum, class, order, and family levels. Sequence data from three individuals were combined.

The compositions of the methanogenic communities as determined by amplicon sequencing of *mcrA* genes are presented in Figure 4. Krona graphs showing the individual *mcrA* compositions are shown in the Supplementary Material (Figure S2). The rumen methanogenic community was dominated by the genera *Methanobrevibacter* (order Methanobacteriales) and the candidate genus *Methanoplasma* (order Methanomassiliicoccales), which together accounted for 85% of all *mcrA* reads. Minor percentages of the sequences were assigned to Methanococcales and Methanomicrobia. Differently from the rumen, *Methanocorpusculum* (order Methanomicrobiales) was the most abundant genus in the manure community with 66%, while only 5% of the manure sequences were assigned to *Candidatus Methanoplasma*. Compared to the bacterial communities, the methanogenic community profiles showed a much higher variance between the three individuals but still formed distinct compartment-specific clusters in the NMDS plot (Figure 3b). The genera *Methanobacterium, Methanobrevibacter*, and *Methanomicrobium* have been described as characteristic hydrogenotrophic rumen methanogens [6]. In accordance with our results, *Methanobrevibacter* was also found as the dominant genus among the rumen methanogens [38,39]. The other abundant genus in the rumen samples, *Candidatus Methanoplasma*, has been described as an H_2_-dependent methylotrophic methanogen [40].

As shown in Table 1, the rumen methanogenic communities had higher OTU numbers as well as higher Chao1 and Shannon indices compared to the manure samples. Compared to the bacterial communities, the methanogenic communities displayed a more uneven species distribution, and the manure communities were more uneven than the rumen communities.

### 3.3. Functional Implications of the Differences in Rumen and Manure Microbiomes

The main function of the rumen microbiome is the hydrolysis and fermentation of plant polymers, resulting in the formation of short chain fatty acids with CO_2_ and H_2_ arising as by-products. While fatty acids are consumed by the ruminant, rumen methanogens use CO_2_ and H_2_ to produce CH_4_. Thus, they act as a hydrogen sink, thereby supporting the activity of fermentative bacteria. Moreover, post decomposition of cellulosic compounds occurs in the large intestine and the colon in foregut ruminants [41]. The digestate passes through the large intestine where water is absorbed and eventually remainders are excreted. Thus, the microbial community of manure reflects the large intestine’s microbiome including the same taxa generally found across the gastrointestinal tract of cattle. However, the abundances of these taxa change across the digestive tract [42]. It has been suggested that the lignocellulolytic bacteria in the residual feed may be involved in downstream fermentation in the lower part of the digestive tract [43]. However, methane is mainly produced in the rumen, whereas only a small portion (11%) of the total methane is formed in the lower part of the digestive system [44].

The microorganisms in the rumen excrete specialized enzymes for lignocellulose degradation. When rumen fluid is used as an inoculum for anaerobic digesters treating lignocellulosic biomass, this adds hydrolytic activity due to the capability of the microbial community to produce the necessary enzymes [45]. Several studies have shown that adding rumen fluid has a positive effect on the performance of anaerobic digesters treating lignocellulosic biomass [7,46].

## 4. Conclusions

The results of our study revealed substantial differences in the bacterial communities of rumen and manure microbiomes with regard to the hydrolytic bacteria known to be involved in plant fiber degradation. Although Bacteroidetes was a dominant phylum in both environments, the families Prevotellaceae and Fibrobacteraceae, both of them dominant in the rumen and known as key cellulose degraders, were not detected in the manure samples, whereas the latter contained a higher proportion of hydrolytic bacteria assigned to the Ruminococcaceae family. Additionally, the methanogenic communities in both environments showed different profiles. The findings of this study provide insights to the rumen microbiome that can be used as seed for anaerobic digesters treating lignocellulosic feedstock due to the high abundance of lignocellulose-degrading bacteria. Although cattle manure is a suitable inoculum for anaerobic digesters providing hydrogenotrophic methanogens and hydrolytic/fermenting bacteria of the orders Clostridiales and Bacteroidales, rumen fluid might be more effective by introducing plant fiber-degrading specialists. However, rumen fluid is not as readily available as manure and therefore inoculation or bioaugmentation with rumen microbiota is difficult on a practical scale. An option to overcome this limitation could be the enrichment and propagation of high-performance lignocellulolytic consortia from rumen samples for inoculation or bioaugmentation with the aim of enhancing biogas production in anaerobic digesters treating lignocellulosic feedstock.

## Figures and Tables

**Table 1:** Summary of the estimated richness and evenness of the bacterial and methanogenic communities of both compartments. Diversity indices were calculated with the QIIME pipeline, except the Shannon index, which was calculated according to Lucas et al. [37].

Sample	No. of Reads	No. of OTUs	Chao1	Shannon	Simpson	Pielou’s Evenness
Rumen bacteria	6998	217	293	5.26	0.99	0.92
Manure bacteria	12,232	402	513	5.89	0.99	0.94
Rumen methanogens	9906	190	292	4.09	0.97	0.76
Manure methanogens	7915	91	161	2.39	0.78	0.51

## Supplementary Materials

The following are available online at http://www.mdpi.com/2076-2607/6/1/15/s1.

Supplementary File 1
