# Supplementary material for: Comparison of Rumen and Manure Microbiomes and Implications for the Inoculation of Anaerobic Digesters
Source: Microorganisms. 2018 Feb 14;6(1):15. doi: 10.3390/microorganisms6010015 (PMC5874629; doi:10.3390/microorganisms6010015)
Supplement: Supplementary File 1 [file microorganisms-06-00015-s001.pdf]

microorganisms

MDPI

Supplementary Material

Comparison of rumen and manure microbiomes and implications for the inoculation of anaerobic digesters

Emine Gozde Ozbayram[1,3], Orhan Ince[1], Bahar Ince[2], Hauke Harms[3], Sabine Kleinsteuber[3,*]

1. Department of Environmental Engineering, Faculty of Civil Engineering, Istanbul Technical University, Maslak, 34469 Istanbul, Turkey; gozbayram@itu.edu.tr, inceor@itu.edu.tr
2. Institute of Environmental Sciences, Boğaziçi University, Bebek, 34342 Istanbul, Turkey; bahar.ince@boun.edu.tr
3. Department of Environmental Microbiology, Helmholtz Centre for Environmental Research - UFZ, 04318 Leipzig, Germany; hauke.harms@ufz.de, sabine.kleinsteuber@ufz.de

* Correspondence: sabine.kleinsteuber@ufz.de; Tel.: +49-341-235-1325

Supplementary Figures:

Figure S1. Krona charts illustrating the bacterial community composition of (a) the rumen samples and (**b**) the manure samples from three individuals on phylum, class, order and family levels.

Figure S2. Krona charts illustrating the methanogenic community composition of (**a**) the rumen samples and (**b**) the manure samples from three individuals on phylum, class, order and family levels.

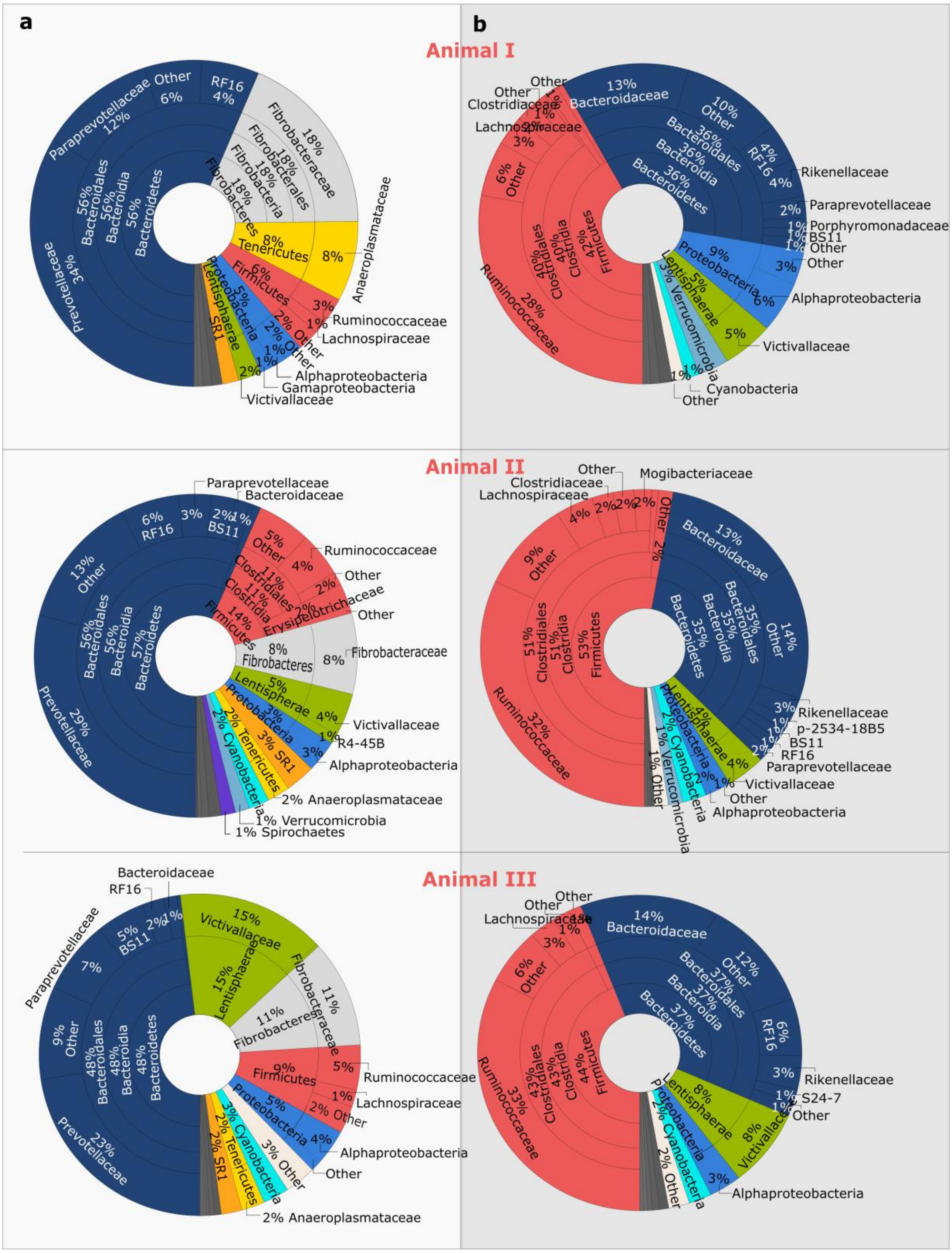

Figure S1. Krona charts illustrating the bacterial community composition of (a) the rumen samples and (**b**) the manure samples from three individuals on phylum, class, order and family levels.

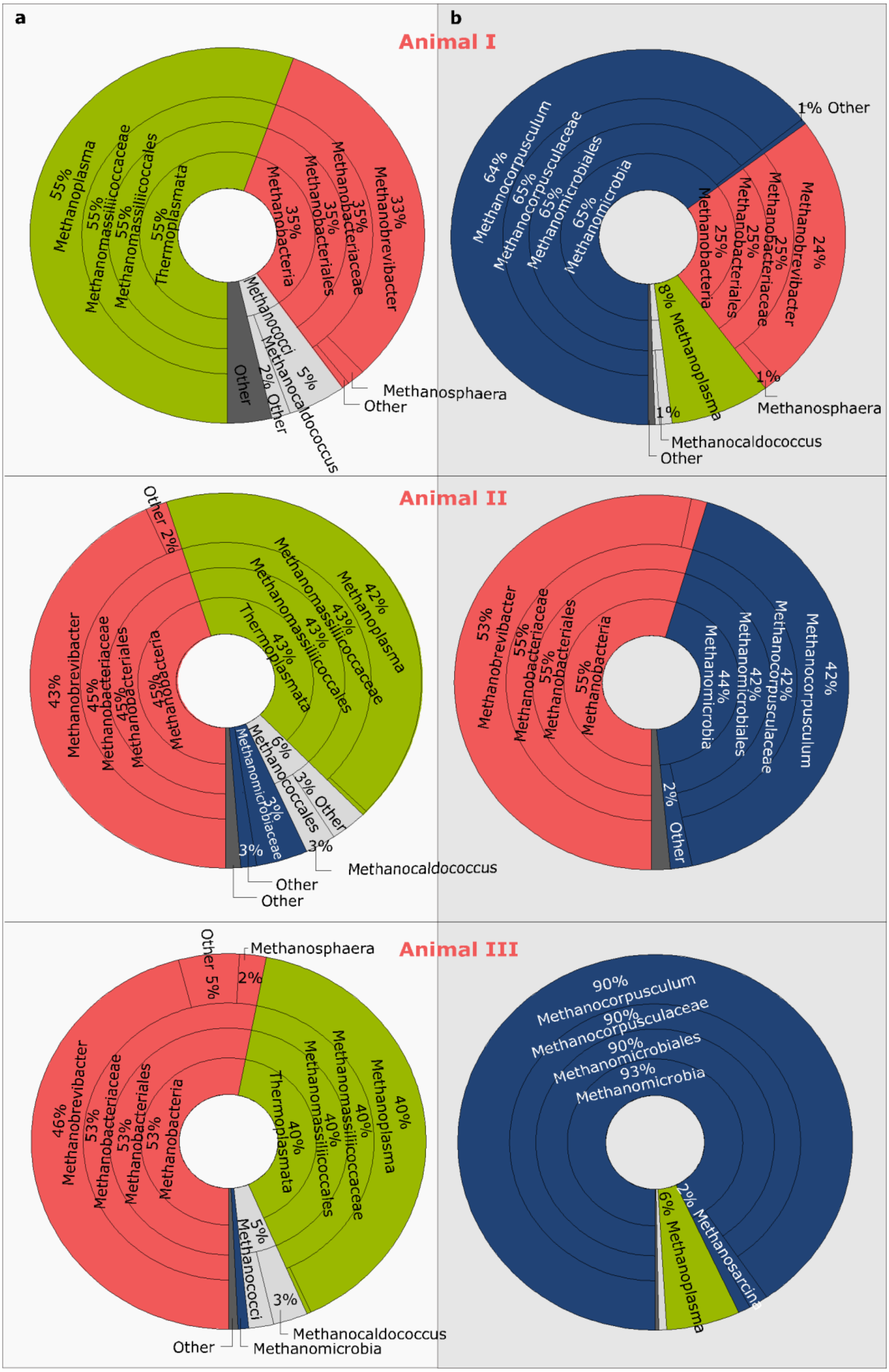

Figure S2. Krona charts illustrating the methanogenic community composition of (**a**) the rumen samples and (**b**) the manure samples from three individuals on phylum, class, order and family levels.
